# Pulmonary Hamartoma Associated With Lung Cancer (PHALC Study): Results of a Multicenter Study

**DOI:** 10.1007/s00408-021-00460-8

**Published:** 2021-07-24

**Authors:** Alfonso Fiorelli, Antonio D’Andrilli, Annalisa Carlucci, Giovanni Vicidomini, Giacomo Argento, Beatrice Trabalza Marinucci, Francesco Ardissone, Roberta Rapanà, Simona Sobrero, Paolo Carbognani, Luigi Ventura, Giovanni Bocchialini, Mark Ragusa, Valentina Tassi, Francesco Sollitto, Domenico Loizzi, Nicoletta Pia Ardò, Marco Anile, Francesco Puma, Erino Angelo Rendina, Federico Venuta, Nicola Serra, Mario Santini

**Affiliations:** 1 Thoracic Surgery Unit, University of Campania Luigi Vanvitelli, Via Pansini, 7, I-80138 Naples, Italy; 2 Thoracic Surgery, Sant’Andrea Hospital, Università La Sapienza, Rome, Italy; 3 Department of Oncology, Thoracic Surgery Unit, San Luigi Hospital, University of Torino, Orbassano, Italy; 4 Medicine and Surgery, Thoracic Surgery, University Hospital of Parma, Parma, Italy; 5 Division of Thoracic Surgery, S. Maria di Terni Hospital, University of Perugia Medical School, Terni, Italy; 6 Thoracic Surgery, Università di Foggia, Foggia, Italy; 7 Department of Thoracic Surgery, Policlinico Umberto I, University of Rome La Sapienza, Rome, Italy; 8 Thoracic Surgery, Università di Perugia, Perugia, Italy; 9 Statistic Unit, Department of Public Health, University of Federico II, Naples, Italy

**Keywords:** Pulmonary, Hamartoma, Surgical resection, Lung cancer

## Abstract

**Purpose:**

Pulmonary hamartoma is the most common benign tumor of the lung. We analyzed a 20-year historical series of patients with pulmonary hamartoma undergoing surgical resection, aiming to evaluate the characteristics, the outcomes, and the association between hamartoma and lung cancer.

**Methods:**

It was a retrospective multicenter study including the data of all consecutive patients with pulmonary hamartoma undergoing surgical resection. The end-points were to evaluate: (i) the characteristics of hamartoma, (ii) outcomes, and (iii) whether hamartoma was a predictive factor for lung cancer development

**Results:**

Our study population included 540 patients. Upfront surgical or endoscopic resection was performed in 385 (71%) cases while in the remaining 155 (29%) cases, the lesions were resected 20 ± 3.5 months later due to increase in size. In most cases, lung sparing resection was carried out including enucleation (*n* = 259; 48%) and wedge resection (*n* = 230; 43%) while 5 (1%) patients underwent endoscopic resection. Only two patients (0, 2%) had major complications. One patient (0.23%) had recurrence after endoscopic resection, while no cases of malignant degeneration were seen (mean follow-up:103.3 ± 93 months). Seventy-six patients (14%) had associated lung cancer, synchronous in 9 (12%) and metachronous in 67 (88%). Only age > 70-year-old (*p* = 0.0059) and smokers > 20 cigarettes/day (*p* < 0.0001) were the significant risk factors for lung cancer.

**Conclusion:**

PH was a benign tumor, with no evidence of recurrence and/or of malignant degeneration after resection. The association between hamartoma and lung cancer was a spurious phenomenon due to common risk factors.

## Introduction

Pulmonary hamartoma (PH) is the most common benign tumor of the lung, accounting for 3% of all lung tumors and having an incidence of 0.25% in the general population [1–5]. PH has been initially considered a developmental malformation, but at present it is classified as a true benign mesenchymal tumor [6–9]. Generally, PH is discovered during assessment for other diseases [10–16], and presents as a solitary pulmonary nodule. Controversies still exist regarding the indication and timing of surgery, the recurrence or malignant degeneration and whether PH is a real risk factor for lung cancer development.

In this study, we analyzed a 20-year historical series of patients with PHs undergoing surgical resection, aiming to evaluate the characteristics, the outcomes, and the association between PH and lung cancer.

## Materials and Methods

### Study Design

This was a retrospective multicenter study. The data of all consecutive patients with PH undergoing surgical resection from January 2000 to May 2020 were analyzed; patients with incomplete follow-up were excluded. The main end-points were to evaluate: (i) the characteristics of PH; (ii) the outcomes, including recurrence or malignant degeneration; and (iii) whether PH could be a risk factor for lung cancer. 

The study was approved by Local Ethics Committee of University of Campania Luigi Vanvitelli (code number: 17402-20), the coordinating center of the study, and then approved by each participating center. 

### Patient Data

The following medical and surgical records were reviewed: age at diagnosis; gender; history of tobacco use; previous, concurrent, and subsequent neoplasms; clinical symptoms; location and size of hamartoma; radiological characteristics of PH; rapidity of growth at Chest Computed Tomography (CT) scan; rational for resection; type of resection; histological features, post-operative outcome, and follow-up. For patients with associated lung cancer, the following data were also collected: time interval between diagnosis of PH and lung cancer, site of lung cancer, stage, histology, and treatment modality. 

### Statistical Analysis 

Variables were reported as mean ± standard deviation (SD), or median and interquartile range for continuous variables, or as number and percentages for categorical variables. Differences between continuous or categorical variables were evaluated by *t*-test or chi-square test, respectively. Logistic regression analysis was performed to identify prognostic factors influencing the presence of lung cancer (dependent variable). The variables reaching statistically significant difference entered a multivariate regression analysis with forward selection and backward elimination. A *p* < 0.05 was considered statistically significant. We used MedCalc statistical software (Version 12.3, Broekstraat 52; 9030 Mariakerke; Belgium) for analyses.

## Results

In the study period a total of 555 patients underwent surgical resection of PH; 15 of these were excluded from the analysis due to incomplete follow-up; thus, our study population included 540 patients (Table 1). The median age was 61.4 [18–85] year-old, with a peak age at diagnosis between the sixth and the seventh decade (38%); 319 (59%) were male, and 91% were smokers (≥ 20 cigarettes a day). At presentation, only 3% of patients (*n* = 15) had specific symptoms as hemoptysis and pneumonia due to endobronchial hamartoma while the most of patients 97% (*n* = 525) had chest symptoms (i.e., coughing, expectoration, and thoracalgia) due to other diseases (444; 82%) or were asymptomatic (81; 15%). The mean diameter of lesion was 18.8 ± 12.4 mm; 98 (19%) patients had tumor larger than 3 cm; 171 (32%) had tumor ≤ 1 cm; in 230 (42%) the tumor size ranged from 10 to 20 mm; and in 230 (42%) from 20 to 30 mm. On CT scan, in most of cases the tumor appeared as a solid lesion, with clear edges and a mean Hounsfield Units of 68 ± 13; in the remaining 112 (21%) cases, the tumor had higher mean HU (215 ± 34) due to the presence of calcification. 18Fluoro-deoxyglucose positron emission tomography-computed tomography (FDG PET-CT) was performed in 160 (30%) patients. The mean maximum standard uptake value (SUV max) was 1.8 ± 0.88, and in 33 (6%) patients was higher than 2.5 (mean value: 3 ± 0.9). The hamartoma was intraparenchymal in 525 (97%) patients and endobronchial in 15 (3%). No patient had multiple lesions. The tumors were evenly distributed throughout the right and left lungs with a frequency approximately proportional to the volume contribution of each lobe as follows: Right Upper Lobe (*n* = 111;21%); Middle Lobe (*n* = 41;7%); Right lower lobe (*n* = 193;26%); Left Upper Lobe (*n* = 130;24%); and Left Lower Lobe (*n* = 119;22%). Before resection, 78 (14%) patients underwent CT-fine needle biopsy (FNAB). It was diagnostic in 49 (62%) cases, not diagnostic in 28 (36%); and positive for malignancy in 1 (2%) case, which turned out to be a hamartoma after excision. Bronchoscopy diagnosed hamartoma in 13 out of 15 (87%) patients with bronchial obstruction.

**Table 1:** Characteristics of study population (*n* = 540)

Variables	All patients (*n* = 540)	Upfront Surgery (*n* = 385)	Surveillance (*n* = 155)	*p*
Age (year-old; median)	61.4 [18–85]	61.2 [18–85]	61.4 [38–74]	0.71
20–30	8 (1%)	5 (1%)	3 (1%)	0.57
31–40	12 (2%)	8 (2%)	4 (3%)	0.72
41–50	80 (15%)	55 (15%)	25 (16%)	0.58
51–60	150 (28%)	100 (26%)	35 (23%)	0.15
61–70	180 (33%)	140 (36%)	30 (20%)	0.002
> 70	110 (21%)	77 (20%)	58 (37%)	0.001
Smokers	493 (91%)	380 (99%)	113 (73%)	0.001
Sex (male)	319 (59%)	232 (63%)	87 (50%)	0.45
Previous comorbidity				
Diabetes	81 (15%)	65 (17%)	16 (10%)	0.05
Hypertension	115 (21%)	80 (21%)	35 (22%)	0.64
Cardiac	120 (22%)	81 (21%)	39 (25%)	0.29
Cerebral	27 (5%)	20 (5%)	7 (4%)	0.74
BPCO	220 (41%)	160 (41%)	60 (39%)	0.54
Neoplastic	67 (12%)	60 (15%)	7 (4%)	0.004
Symptoms				
None	81 (15%)	45 (12%)	35 (22%)	0.002
Cough	220 (41%)	180 (47%)	40 (26%)	< 0.0001
Thoracalgia	79 (14%)	50 (13%)	29 (19%)	0.07
Expectoration	145 (27%)	99 (26%)	46 (30%)	0.04
Hemoptysis	13 (2%)	13 (34%)	0 (0%)	0.02
Pneumonia	2 (0,3%)	2 (0.5%)	0 (0%)	0.30
Pyrexia	27 (5%)	16 (4%)	11 (7%)	0.15
Weight loss	31 (6%)	20 (5%)	11 (7%)	0.39
Size mm (mean)	18.8 ± 12.4	19.3 ± 7.4	10 ± 3.2	0.003
≤ 10 mm	171 (32%)	51 (13%)	120 (77%)	< 0.0001
> 10–20 mm	230 (42%)	202 (52%)	28 (18%)	< 0.0001
> 20–30 mm	41 (7%)	36 (9%)	5 (3%)	0.01
> 30 mm	98 (19%)	96 (25%)	2 (1%)	< 0.0001
CT findings				
Calcification	112 (21%)	12 (3%)	100 (64%)	< 0.0001
Fat	37 (7%)	7 (2%)	30 (19%)	< 0.0001
Hounsfield Units	68.9 ± 12.4	66.3 ± 9.4	212 ± 57	< 0.0001
PET	160 (30%)	127 (33%)	33 (21%)	0.007
Mean SUV	1.8 ± 0.88	1.89 ± 0.6	1.0 ± 0.3	0.001
SUV > 2.5	33 (6%)	30 (8%)	3 (2%)	0.01
SUV ≥ 1.5 ≤ 2.5	97	90 (23%)	7 (4%)	< 0.0001
SUV < 1.5	30	7 (2%)	23 (15%)	0.0009
Preoperative FNAB	78 (14%)	33 (8%)	45 (29%)	< 0.0001
Diagnostic	49 (62%)	4 (1%)	45 (29%)	< 0.0001
Inconclusive	28 (36%)	28 (7%)	0 (0%)	0.0006
Positive for malignancy	1 (2%)	1 (0.2%)	0 (0%)	0.52
Preoperative bronchoscopy	15 (3%)	15 (4%)	0 (0%)	0.01
Diagnostic	13 (87%)	13 (3%)	0 (0%)	–
Inconclusive	2 (13%)	2 (0.5%)	0 (0%)	–
Localization				
RUL	111 (21%)	79 (20%)	32 (21%)	0.97
ML	41 (7%)	30 (8%)	11 (7%)	0.78
RLL	139 (26%)	99 (26%)	40 (26%)	0.98
LUL	130 (24%)	93 (24%)	37 (24%)	0.94
LLL	119 (22%)	84 (22%)	35 (22%)	0.89
Surgical resection	535	380 (99%)	155 (100%)	0.15
Enucleation	256 (47%)	173 (45%)	83 (53%)	0.07
Wedge resection	228 (43%	160 (41.8%)	68 (44%)	0.62
Lobectomy	43 (7.9%)	41 (11%)	2 (1%)	0.0003
Bilobectomy	1 (0.1%)	1 (0.2%)	0 (0%)	0.52
Segmentectomy	7 (1%)	5 (1%)	2 (1%)	0.99
Endoscopic resection	5 (1%)	5 (1%)	0 (0%)	0.15

### Treatment

Upfront surgical (*n* = 380) or endoscopic resections (*n* = 5) were performed in a total of 385 (71%) cases. In comparison with surveillance patient group, the decision of upfront surgery or endoscopy was based due to (i) the presence of specific symptoms due to airway obstruction (15 vs. 0; *p* = 0.01); (ii) the higher risk of the lesion to be malignant based on patients’ history of previous malignancy (15% vs. 4%; *p* = 0.004), solid pattern on CT scan without calcification (3% vs. 64%; *p* < 0.0001) and/or of fat tissue (2% vs. 19%; *p* < 0.0001), uptake on PET scan (1.89 ± 0.6 vs. 1.0 ± 0.3; *p* = 0.01), and not diagnostic CT-guided FNAB (7% vs. 0%; *p* = 0.0006); (iii) the presence of synchronous lung cancer (*n* = 9; 3% vs. 0%; *p* = 0.05); and (iv) the decision of patients who were uncomfortable with adopting a strategy of surveillance, despite the lesion presented a low risk of malignancy (i.e., presence of calcification and/or fat on CT scan, lack of uptake on PET scan, diagnosis of benignity after FNAB) (*n* = 21; 6%). In the remaining 155 (29%) cases, the lesions had low risk of malignancy based on the presence of calcification and of fat on CT scan, no-uptake on PET, and diagnosis of benignity on FNAB findings. Thus, these patients were followed with serial CT scan (mean follow-up: 20 ± 3.5 months), and the decision for surgery was made due to increasing in size of tumor in the follow-up (mean increase size: 10 ± 2.9 mm). In most cases, lung sparing resection was carried out by enucleation (*n* = 256; 47%) and wedge resection (*n* = 228; 43%). Anatomic pulmonary resections were performed in 51 patients with hilar PH or with lung cancer and associated PH. Lobectomy was performed in 43 (7.8%) patients associated with partial bronchoplastic resection in 3 cases; segmentectomy in 7 (1%); and bilobectomy in 1 (0,2%). The procedures were performed via thoracotomy (*n* = 160; 27%); and VATS (*n* = 375; 73%). In 21 (6%) cases, VATS was converted to thoracotomy due to the difficulty in detecting the lesion. All of the three main tissue types including mature hyaline cartilage, fibromyxoid stroma, and mature adipose tissue were present in every individual lesion, but mature hyaline cartilage represented the major constituent (more than 50%) in 282 out of 540 (52%) tumors.

### Outcome and Follow-up

The results were summarized in Table 2. No complications occurred during surgery. The length of chest drainage and of hospital stay was 4.0 ± 2.6 days and 5.5 ± 2.8 days, respectively. Twenty/two (4%) patients presented postoperative complications occurring after lobectomy (*n* = 20; 91%), enucleation (*n* = 1; 4.5%), and wedge resection (*n* = 1; 4.5%). Thirteen (2.4%) patients had persistent air-leaks that resolved spontaneously in all but one which was successfully treated with endobronchial valves; 7 (1.3%) had atrial fibrillation; and 2 (0.3%) had hemothorax requiring surgical exploration by thoracotomy.

**Table 2:** Surgical outcome in patients undergoing lung resection (*n* = 535)

Variables	All patients (*n* = 535)	Thoracotomy (*n* = 181)	VATS (*n* = 354)	*p*-value
Type of resection				
Enucleation	256 (47%)	83 (46%)	173 (49%)	0.50
Wedge resection	228 (43%	67 (37.5%)	161 (45.5%)	0.06
Lobectomy	43 (7.9%)	25 (14%)	18 (5%)	0.0004
Bilobectomy	1 (0.1%)	1 (0.5%)	0	0.16
Segmentectomy	7 (1%)	5 (3%)	2 (0.5)	0.03
Length of chest drainage (days)	4.0 ± 2.6	7.3 ± 3.8	3.4 ± 1.8	0.007
Length of hospital stay (days)	5.5 ± 2.8	8.9 ± 2.9	4.3 ± 2.1	0.005
Complications	22 (4%)	10 (5%)	12 (3%)	0.23
Persistent air-leaks	13 (2%)	6 (3%)	7 (2%)	
Atrial Fibrillation	7 (1%)	4 (2%)	3 (1%)	
Hemothorax	2 (0.3%)	0	2 (0.5%)	

Thoracotomy compared to VATS group was associated with higher incidence of anatomical resection including lobectomy (14% vs. 5%, *p* = 0.0004) and segmentectomy (3% vs.0.5%; *p* = 0.03), longer length of chest drainage (7.3 ± 3.8 vs. 3.4 ± 1.8; *p* = 0.007), and of hospital stay (8.9 ± 2.9 vs. 4.3 ± 2.1; *p* = 0.005), but no significant difference was found regarding post-operative morbidity (5% vs. 3%; *p* = 0.23). The mean follow-up time was 103.3 ± 93 months [1–370 months]. Only one patient (0.2%) had recurrence 11 months later endoscopic resection; he underwent a second resection via rigid bronchoscope and no further recurrence occurred. 

### Cancer Associated to Hamartoma

**Fig. 1:**
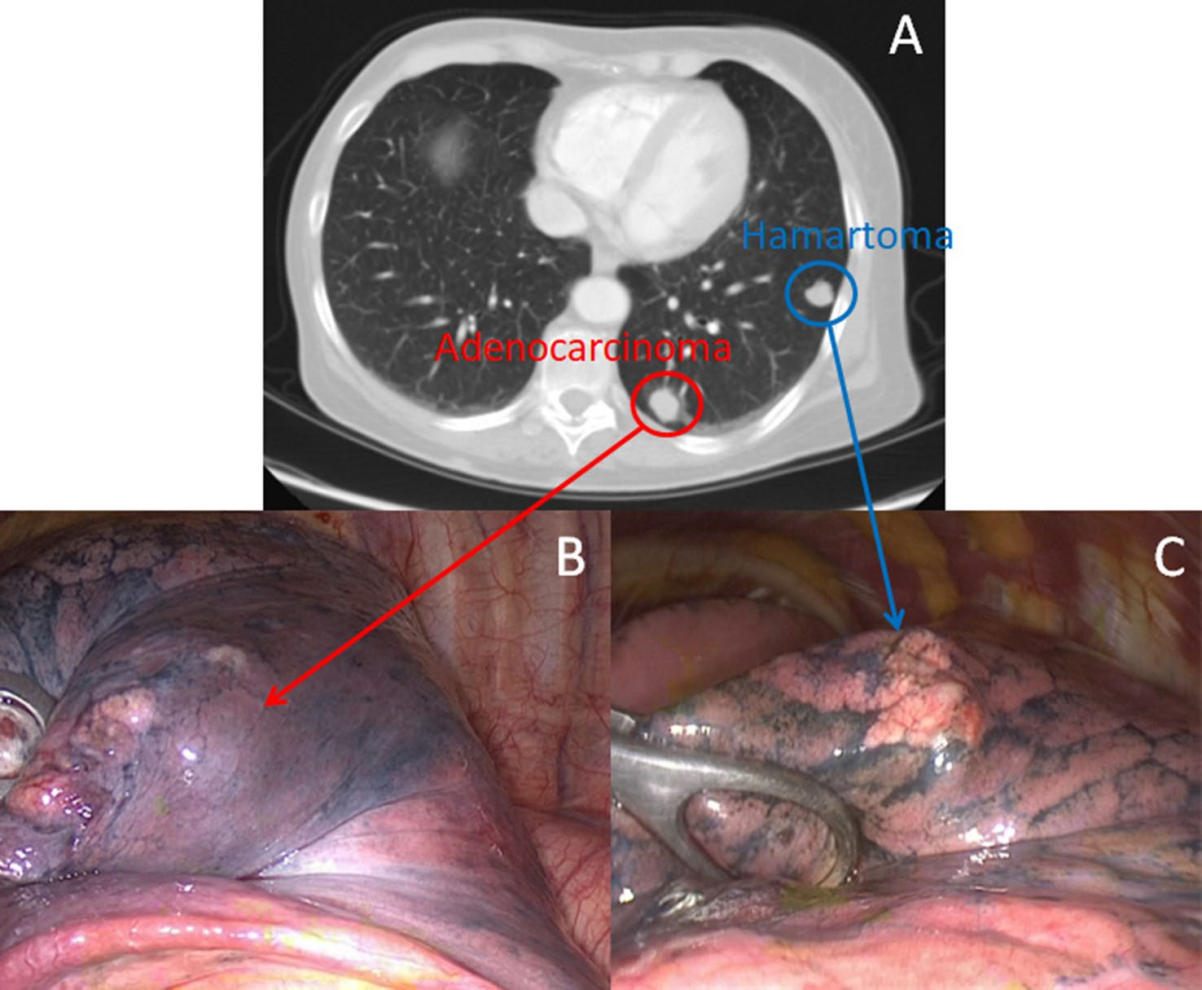
A 57-year-old woman had hamartoma and adenocarcinoma in the left lower lobe. She underwent thoracoscopy left lower lobectomy. Part **A** = CT scan; Part **B** and Part **C** = operative view of adenocarcinoma (B) and hamartoma (C)

One hundred forty-three out of 540 (26%) patients had associated cancer (Table 3). Sixty-seven (12%) out of 540 patients had associated extrapulmonary malignancies. In one case (1%), the diagnosis of PH was synchronous, in 60 (91%) subsequent, and in 6 (8%) antecedent to the diagnosis of the extrapulmonary malignancies. Seventy-six (14%) patients had associated lung cancer; it was synchronous in 9/76 (12%) (Fig. 1), and metachronous in 67/76 (88%) patients. Most patients with associated lung cancer were male (56%), and all but two were smokers (97%). The mean interval time between the diagnosis of hamartoma and lung cancer was 20 ± 4.5 months. Twenty-eight lesions (36%) involved the ipsilateral lung, and 20 of these (26%) the same lobe of the hamartoma; while 48 (64%) the contralateral lung of the hamartoma. The mean size of hamartoma was 17 ± 9.3 mm; it had a shallow margin in 50 cases (66%), and mature hyaline cartilage represented the major constituent in 41 (54%) cases. Hamartoma was resected in association with synchronous lung cancer by lobectomy in 6 cases (8%), by enucleation in 55 (72%), and by wedge resection in 24 (20%). For resection of lung cancer, lobectomy was performed in 60 patients (79%), and segmentectomy in 16 patients (21%) due to limited respiratory function.

**Table 3:** Malignancy associated with PH (n = 143)

Variables	Total number	Synchronous	Metachronous	
			Antecedent	Subsequent
*Lung Cancer*				
Number	76 (53%)	9 (12%)	–	67 (41%)
Age	63.4 ± 9.8	60 ± 8.7	–	63.1 ± 7.8
Sex (male)	43 (56%)	7 (9%)	–	36 (47%)
Smokers	74 (97%)	9 (12%)	–	65 (85%)
Interval between hamartoma and tumor (months)	20 ± 4.5	–		20 ± 4.5
Site				
Ipsilateral	8 (10%)	3 (3%)	–	5 (7%)
Same lobe	20 (26%)	6 (8%)	–	14 (18%)
Contralateral	48 (64%)	0	–	48 (62%)
Histology				
Adenocarcinoma	47 (62%)	5 (7%)	–	42 (55%)
Squamous cell carcinoma	26 (34%)	4 (5%)	–	22 (29%)
Large Cell carcinoma	3 (4%)	0	–	3 (4%)
pStage				
Stage I	53 (70%)	7 (10%)	–	46 (60%)
Stage II	19 (25%)	2 (3%)	–	17 (22%)
Stage III	4 (5%)	0	–	4 (5%)
Type of resection (hamartoma/lung cancer)				
Lobectomy	6 (8%)	6 (8%)	–	0
Enucleation + Lobectomy	45 (59%)	3 (4%)	–	42 (55%)
Wedge resection + Lobectomy	9 (12%)	0	–	9 (12%)
Wedge resection + segmentectomy	16 (21%)	0	–	16 (21%)
*Extrapulmonary Malignancy*	67 (47%)	1 (1%)	60 (42%)	6 (4%)
Breast	17 (26%)	0	15 (22%)	2 (4%)
Thyroid	2 (3%)	0	2 (3%)	0
Gastric	10 (15%)	0	10 (15%)	0
Colon	14 (21%)	0	13 (19%)	1 (2%)
Kidney	15 (22%)	1 (1%)	12 (18%)	2 (3%)
Lymphoma	5 (7%)	0	5 (7%)	0
Skin	4 (6%)	0	3 (5%)	1 (1%)

Logistic regression analysis reported in Table 4 found that only age > 70 years (odds ratio: 0.469; *p* = 0.0059) and smokers more than 20 cigarettes/day (odds ratio:5.083; *p* < 0.0001) were significant risk factors for cancer development.

**Table 4:** Logistic regression analysis for development of lung cancer

Variables	Odds Ratio	95% CI	*p*-value
Age > 70 years (yes/not)	0.469	0.273 to 0.803	0.0059
Sex (male/female)	1.447	0.836 to 2.50	0.18
History of cancer (yes/not)	1.706	0.838 to 3.47	0.14
Smokers more than 20 cigarettes/day (yes/not)	5.083	3.001 to 8.60	< 0.0001
Size ≥ 30 mm (yes/not)	1.018	0.492 to 2.10	0.96
SUV > 2.5 on PET scan (yes/not)	2.057	0.812 to 5.21	0.12
Smooth margin (yes/not)	0.817	0.449 to 1.48	0.50
Calcification (yes/not)	1.205	0.639 to 2.27	0.56
Increase in size during follow-up (yes/not)	1.358	0.871 to 2.11	0.17
Hamartoma chondroma histology (yes/not)	0.877	0.373 to 2.06	0.76

## Discussion

The term “hamartoma” derives from the Greek word and means “error”. It was firstly used in 1904 by Albrecht [17] to define tumor resulting from an error in development of tissues normally present in the involved organ. In 1934, Goldsworthy defined PH as a benign tumor composed of a combination of fat and cartilage [1, 2]. The clinical significance of PH is still debate with concerns on malignant degeneration or association with lung cancer. To define these issues, we planned a multicenter study collecting the largest number of patients with PH undergoing surgical resection published so far.

**Fig. 2:**
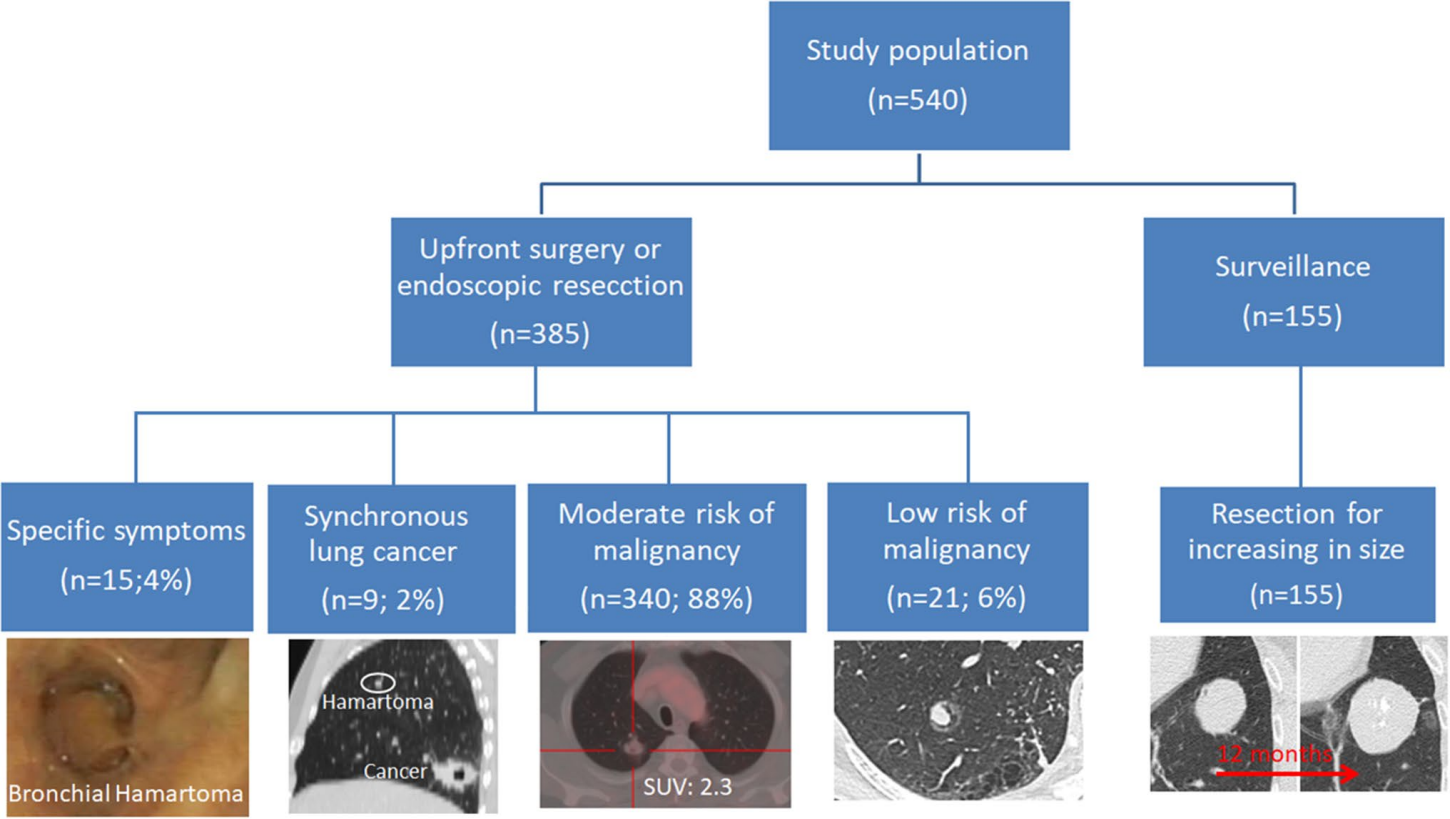
Algorithm for decision making

First, in line with data of literature, in the most of our cases PH was incidentally discovered as peripheral nodule on CT scan performed for other diseases. A small number of lesions presented calcifications or fat on CT scan and were FDG avid. In 36% of cases, the CT-FNAB results were not diagnostic for the difficulty in aspirating adequate materials due to the dense structure of the lesion [18, 19]. The most of our patients underwent upfront surgical. As summarized in Fig. 2, the decision was dictated (i) by the presence of specific symptoms related to tumor, (ii) by the higher risk of the lesion to be malignant based on clinical and radiological findings, (iii) by the presence of synchronous cancer, or (iv) by decision of patients who were uncomfortable with adopting a strategy of surveillance, despite the lesion radiologically seemed to be benign for the presence of calcification or of fat on CT scan, or the lack of FDG uptake [20]. In the remaining patients with low risk of cancer, the tumor was followed up with planned CT scan, and then resected due to increasing in size. Lung sparing resections by VATS were the main surgical strategy, while anatomic resections were performed only in selected cases due to tumor extension. VATS compared to thoracotomy was associated with shorter hospitalization but similar post-operative complications, thus the different extend of resections rather than the surgical approach likely affected these results as the most of anatomic resections were performed by thoracotomy.

Second, the outcome observed in our series confirmed the benign nature of tumor and excluded malignant transformation or recurrence. Only one patient (0.2%) had tumor recurrence 11 months after incomplete endoscopic resection, while no case of malignant transformation was found. Our results were in line with previous series published in the English literature [1–16] and considered collectively with the present in Table 5. Among 1,733 patients evaluated, recurrence was found in only 4 cases (0.23%) after enucleation in the same site of previous excision, supporting that a R0 resection was mandatory to prevent recurrence. No cases of malignant transformation were found, confirming the unfeasibility of this event. Despite sporadic single experiences reported malignant degeneration of PH, the evidences supporting this hypothesis were weak [21]. In some papers, hamartoma and carcinoma cell lines seemed to be independent from each other, suggesting a coexistence of the two tumors rather than a degeneration of PH [22]. In others, PH involved the pleura and this unusual growth pattern was considered a sign of malignant transformation, but no malignant cells were detected in the pleura specimens [23]. Furthermore, there was no evidence of malignant transformation in patients not undergoing upfront surgery but followed with planned CT scan in our and in Sinner’s series [24] after a mean follow-up of 2 and 5 years, respectively.

**Table 5:** Review of the literature regarding recurrence and malignant transformation of PH

Authors	No. of PH	Recurrence	Malignant transformation
Koutras et al. [1]	19	0	0
Karasik et al. [2]	52	0	0
Fudge et al. [3]	29	0	0
van Den Bosch et al. [4]	154	2	0
Crouch et al. [5]	19	0	0
Salminen et al. [6]	77	0	0
Hansen et al. [7]	89	0	0
Ribet et al. [8]	65	0	0
Gjevre et al. [9]	216	0	0
Lee et al. [10]	29	0	0
Lien et al. [11]	62	0	0
Guo et al. [12]	39	1	0
Çaylak et al. [13]	20	0	0
Wang [14]	226	0	0
Ekinci et al. [15]	73	0	0
Haberal et al. [16]	24	0	0
Our series	540	1	0
Total	1733	4 (0.23%)	0

Third, the incidence of lung cancer in our study population was 14%, which was higher than that observed in the general Italian population [25]. Similarly, Karasik et al. [2], and Ribet et al. [8] found that the incidence of lung cancer in PH population was higher of 6.3 and 6.7 times than that expected for the general Israeli and French population, respectively. However, the higher incidence of lung cancer in patients with PH did not mean that PH was a real risk factor for lung cancer development. In 42% of all cases reported in literature (Table 6), lung cancer and hamartoma were in the same lobe, excluding any spatial correlation between two tumors as previously supported by Karasik et al. [2]. Furthermore, in our and other series (8, 9), PH and lung cancer were associated with similar risk factors as smoking and advanced age. Thus, the coexistence of two tumors was a spurious event due to the exposure to the same risk factors. Patients with PH should undergo a regular follow-up, especially if they are smokers and elderly, as it could discover a metachronous early-stage lung cancer. Additionally, in 91% of cases (60 out of 67 cases) hamartoma was diagnosed during follow-up for extra thoracic malignancy. Thus, the previous existence of extra thoracic cancer could lead to the discovery of PH that would have remained unknown otherwise.

**Table 6:** Review of literature regarding PH associated with lung cancer

Authors	No. of PH	No. Of Associated Lung Cancer			Same lobe (Lung cancer and PH)
		Total number	Synchronous	Metachronous	
Koutras et al. [1]	19	1 (5.2%)	1 (5.2%)	0	0
Karasik et al. [2]	52	4 (7.6%)	1 (1.9%)	3 (5.7%)	4 (100%)
Fudge et al. [3]	29	5 (17.2%)	1 (3.4%)	4 (13.8%)	N/A
van Den Bosch et al. [4]	154	11 (7.1%)	6 (3.8%)	5 (3.3%)	5 (45.4%)
Crouch et al. [5]	19	2 (10.5%)	2 (10.5%)	0	2 (100%)
Salminen et al. [6]	77	1 (1.2%)	0	1 (1.2%)	N/A
Hansen et al. [7]	89	1 (1.1%)	1 (1.1%)	0	1 (100%)
Ribet et al. [8]	65	3 (4.6%)	0	3 (4.6%)	0
Gjevre et al. [9]	216	45 (1.1%)	39 (18%)	6 (2.8%)	16 (35.5%)
Ekinci et al. [15]	73	17 (23%)	13 (17.8%)	4 (5.2%)	4 (23.5%)
Our series	540	76 (14%)	9 (1.6%)	67 (12.4%)	20 (26%)
Total	1333	166 (12.4%)	73 (5.4%)	94 (7%)	52 (42%)*

*This value was calculated on the total of 1227 cases as in 106 cases it was not reported which lobe was affected by lung cancer

Obviously, our results should be evaluated with caution, before drawing definitive conclusions. Due to the retrospective and multicenter nature of the study, there was not a standardized protocol for the timing, and the rational of the surgery, the histological diagnosis, and the clinical follow-up modality. The lack of a surveillance control group made impossible to know whether all PHs undergoing upfront surgery would remain stable in size over the time. All participants centers were thoracic surgery units, thus the most of patients were referred for a surgical evaluation due to the risk that the lesion was a cancer. Thus, it likely explained the small number of lesions with radiological characteristics of PH, and on the other hand the high number of patients planned for upfront surgery.

In conclusion, PH was a benign tumor, with no evidence of recurrence and/or of malignant degeneration after resection. The presence of PH did not increase the risk of lung cancer development, but the association between two tumors was spurious and due to the exposure to the same risk factors. 
